# Fractal Indices as Estimators of the Complexity of Ephemeroptera Nymph Movement in a Thermal Stress Experiment

**DOI:** 10.1002/ece3.73042

**Published:** 2026-04-09

**Authors:** Jorge Machuca‐Sepúlveda, Mauricio Zamorano, Jorge G. Farías

**Affiliations:** ^1^ Department of Chemical Engineering, Faculty of Engineering and Science Universidad de La Frontera Temuco Chile; ^2^ Facultad de Ciencias Forestales y de la Conservación de la Naturaleza Universidad de Chile Santiago Chile

**Keywords:** Baetidae, fractal dimension, Leptophlebiidae, locomotor traits, lyophilized weight, maximum body length, microhabitat

## Abstract

Global warming increases water temperature of freshwater habitats, altering thermotaxis responses of hemimetabolous macroinvertebrates. Ephemeroptera nymphs are very vagile active in different types of microhabitats. Its movement complexity could shift during temperature augmentation, dynamic that has been received little attention. The present study examined the two‐dimensional and movement behavior complexity of Ephemeroptera nymphs (Baetidae and Leptophlebiidae) under an experiment of induced thermal stress. Increasing temperatures could influence the movement patterns according to Fractal Dimension Index (FDI). FDI methods (radial, box‐counting, and dilation) indicate the complexity of trajectories. In a acclimatized room, recording the movement of nymphs under three thermal conditions: control (temperature from sampling sites), control +5°C and control +10°C, from several microhabitats of rithronic streams, were conducted. An aquarium was continuously aerated with an air pump and covered at night to simulate the natural photoperiod, where nymphs were sheltered after capture. The results showed that thermal stress led to a decrease in FDI, suggesting a shift toward more directional and less complex movement patterns. Compared to families, microhabitats have more significative effect across FDI methods. Nymphs from structurally complex habitats (macrophytes and boulders) tended to maintain higher FDI values under stress, as opposed to those from simpler (gravel and sand), which showed a sharper reduction in movement complexity. Hence, the negative effects of heat stress on behavior may mitigate by structural heterogeneity of habitats. Positive correlations were found between habitat and locomotor traits such as Mean speed and Total path length, suggesting that complex habitats may enhance nymph mobility. Our findings support the use of fractal metrics as indicators of behavioral plasticity and complexity of animal movement, under environmental stress.

## Graphical abstract

The proposed research addresses a topic of great environmental and ecological relevance, emphasizing the sensitivity of freshwater biota to environmental changes, particularly global warming and water temperature augmentation. This context is crucial, since the increase in temperature directly affects metabolic rates, locomotion and spatial distribution of ectothermic organisms such as aquatic hemimetabolous (Ephemeroptera), making it essential to study their movement patterns under thermal stress.

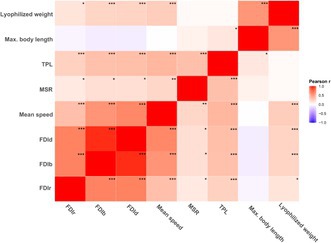

## 1 Introduction

Freshwater biota is highly sensitive to environmental changes, particularly due to global warming, which significantly influences the behavior of ectothermic organisms, for instance, aquatic hemimetabolous insects (Poff et al. 2007). Thermal behavioral effects include kinetic effects, constraining metabolism and neurophysiology bottom‐up; and integrated effects, where thermal information initiates or modifies intentional behavioral responses (Abram et al. 2017). Temperature‐induced changes in their movement complexity may have profound implications for ecological interactions, such as predator–prey dynamics and resource acquisition strategies (Angilletta Jr. 2009; Woodward et al. 2010). Temperature affects metabolic rates, locomotion, and spatial distribution in their habitats, making it crucial to study the movement patterns of these organisms under thermal stress (Dell et al. 2014; Rezende and Bozinovic 2019). Changes in spatial conditions that affect fractal calculations mean changes in habitats, surface areas, or occupation patterns. Density, clustering, and the presence of obstacles also have an influence, which are mainly affected by body length and weight of organisms (Dibble and Thomaz 2009; García‐Gutiérrez et al. 2009). While physiological and population‐level responses of aquatic insects to temperature have received considerable research attention, behavioral responses at the individual level—especially locomotor dynamics—remain underexplored (Bonacina et al. 2023; Dallas and Rivers‐Moore 2012).

Global warming on freshwater ecosystems produce loss and degradation of habitats., By prioritizing habitat preservation, it may be possible to enhance the resilience of these ecosystems against climate change (Carosi 2022). Challenges such as habitat degradation and climate change act as barriers to the relocation strategies of freshwater macroinvertebrates in streams, hindering their conservation and the overall health of river ecosystems (Lim and Do 2023; Smith et al. 2020). The lack of integrative studies combining temperature, habitat structure, and spatial movement metrics represents a significant knowledge gap, key for relocation strategies, for instance.

Among hemimetabolous aquatic insects, mayfly nymphs (Ephemeroptera) display narrow thermal niches and serve as bioindicators of temperature change (Souza et al. 2024). Temperature increases have been shown to influence mayfly physiology and survival thresholds such as Critical Thermal Maxima (CTMax), leading to reduced ventilation and loss of equilibrium at elevated temperatures (Verberk et al. 2016). Mayfly larvae exhibit microhabitat‐specific distributions, often associated with structurally complex microhabitat, such as cobbles, moss, or gravel in streams (Vilenica et al. 2018). The structural characteristics of microhabitats (e.g., cobbles, gravel, macrophytes, detritus) are known to shape mayfly presence and functional diversity (Tokeshi and Arakaki 2012). Recent experimental work with insects revealed that microhabitat heterogeneity buffers the negative effects of heat stress, enabling more stable activity levels, while homogenized microhabitat exacerbate activity declines under warming (Terlau et al. 2023). Habitat degradation—via sedimentation, vegetation loss, or stream warming—can disrupt structural complexity and consequently shape movement paths of behavioral interactions (Ciborowski and Clifford 1984). In addition, environmental heterogeneity at the microhabitat scale plays a crucial role in shaping animal movement behavior, particularly under stress (Dibble and Thomaz 2009). The reduction of structural refugia may exacerbate behavioral stress, leading to altered movement patterns that are more directional and less tortuous—measurable through fractal dimension analysis. Nevertheless, the interaction between microhabitat complexity, thermal stress, and locomotor behavior in Ephemeroptera remains largely unexplored.

The ongoing loss of structurally complex microhabitats due to climate‐induced perturbations and anthropogenic habitat degradation—such as temperature‐driven droughts and sedimentation—exacerbates the vulnerability of stream‐dwelling nymphs (Meerhoff and de los Ángeles González‐Sagrario 2021). In this regard, fractal dimension is a powerful quantitative descriptor of movement complexity (Nams 2006). Higher fractal dimension values typically indicate tortuous, exploratory paths, while lower values correspond to straighter, directional movements (With et al. 1999). Recent methodological advances in fractal analysis combine box‐counting, radial, and dilation methods to more comprehensively describe path structure (Cui and Wang 2025). Among the tools used to quantify behavioral responses, the Fractal dimension index (FDI) has proven effective for analyzing the complexity and irregularity of movement trajectories (Nathan et al. 2008; Seuront 2011). Quantifying movement complexity through fractal analysis has proven valuable in other contexts by revealing nonobvious changes in path structure under stress or altered ecological conditions (e.g., Lévy vs. Brownian patterns in animal foraging). However, this approach has seldom been applied to freshwater insect locomotion under temperature stress (Humphries et al. 2010; Nathan et al. 2008).

Therefore, FDI analysis could offer a simple perspective by quantifying these behavioral modifications in several types of paths, which might otherwise remain undetected using traditional metrics (Seuront 2011). FDI values approaching two indicate highly tortuous, space‐filling trajectories, whereas values near one correspond to straighter, more directional movements (Dicke and Burrough 1988; Turchin 1996). Some examples of studies that applied fractal dimension analysis has been proved in invertebrates (Reynolds et al. 2007; Reynolds and Frye 2007; Uttieri et al. 2005) and vertebrates (Bascompte and Vilà 1997; Eguiraun and Martinez 2023; MacIntosh et al. 2013). Recent studies have highlighted the potential of fractal metrics in behavioral ecology, demonstrating their ability to detect subtle changes in movement patterns driven by environmental stressors (Loke and Chisholm 2022; Suryanto et al. 2022).

Few studies have explicitly connected temperature‐induced changes in fractal dimension to hypotheses about movement complexity. Hence, the hypothesis proposed as follows: (H1) thermal stress (increased water temperature) leads to decreased fractal dimension (FDI) reflecting less complexity in movement behavior of mayfly nymphs, (H2) microhabitat structure modulates the effects of thermal stress on fractal movement patterns, as indicated by the FDI, and (H3) families of mayfly nymphs differ in their locomotor and morphological traits in structurally complex habitats, as revealed by multivariate patterns in FDI, movement behavior, and body characteristics.

Our study attempts to fill these knowledge gaps by analyzing FDI to examine the behavioral complexity of mayfly nymph movement under controlled increases in water temperature. In an experiment conducted in a closed, acclimatized enclosure, nymphs from the Leptophlebiidae and Baetidae families, obtained from rithronic streams in the southwestern Andes, could exhibit different behaviors. The aim is to combine fractal indices with movement behavior variables to provide a comprehensive understanding of how these organisms respond to gradual thermal stress and their respective path modes. These findings are expected to contribute to a broader debate on the impacts of global warming on freshwater ecosystems, relevant to biomonitoring activities (Fierro et al. 2024), and the possible outcomes of mitigation measures, mainly relocation or dispersion patterns.

## 2 Methodology

### 2.1 Sampling Organisms and Experimental Setup

**FIGURE A1:**
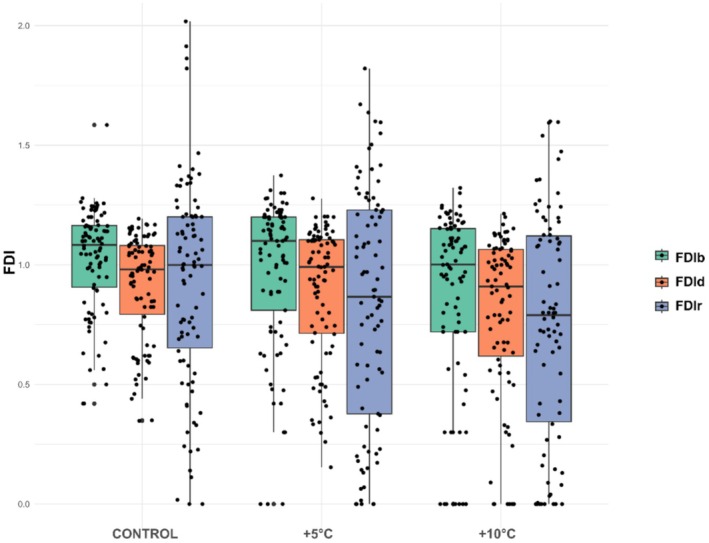
Boxplots of fractal dimension indices (FDI) calculated via three methods (box‐counting = FDIb, dilation = FDId, radial = FDIr) across three thermic stress treatments: Control (natural water temperature), +5°C, and +10 C. Points represent individual nymphs' trajectories. A decrease in FDI values indicates less complex movement.

**FIGURE A2:**
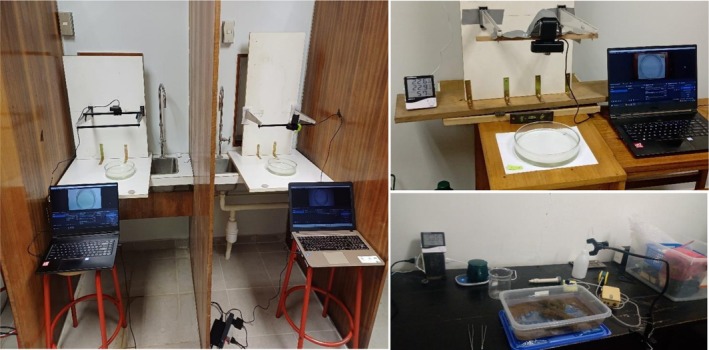
Photography of acclimatized room and aquarium where experiment setup was established. Note the parallel arrangement of the arenas to save time and reduce stress on the nymphs in the aquarium. The video recording system was created directly through a webcam and notebook.

Examined aquatic nymphs (Ephemeroptera: Baetidae and Leptophlebiidae), from streams located in distinct rithronic environments in central‐southern Chile (33° to 38° lat. S), were collected. In several microhabitats, including detritus, macrophytes, inorganic sediments, and boulders (Burgazzi et al. 2021), individuals were sampled. After field collection, specimens were transported to the laboratory and acclimatized in aquaria at natural stream temperatures for 24 h. Later, criteria for selection of individuals is straightforward: body length should be > 0.2 cm, very active in aquaria, without wing pads (intermediate instar), with legs, antennae, and gills intact or without damage, and without algal matter or sediment grains in their body. Locomotion recordings were conducted using a standardized arena (Petri dishes of 20 cm in diameter) filled with filtered and aerated stream water. The arenas contain up to 50% of the total available water content in the Petri dish, that is, approximately 300 mL. The height of the water column in all arenas is 2.8 cm. Each nymph was placed individually in the arena, and its movement was recorded for 600 s under three controlled thermal conditions. Simulating a thermal stress gradient: (1) control (natural water stream temperature, in sampling site), (2) control +5°C, and (3) control +10°C, specimens moved from one arena to another in a linear and irreversible fashion. Thermal treatments were set up by warming water in an electric heater, monitored with thermostats. Given the effective volumetric range of movement, there is uncertainty about the positions that the nymphs will adopt during the recording. Considering that there is only one webcam from above in a zenithal view (90° to the diameter of the arena), it is possible that the nymphs will move across the arena by crawling along the bottom or swimming on the surface or in intermediate areas. Therefore, in the video, they could be seen in different body positions, either just their head (circle) or in ventral/dorsal view (rectangle). For this reason, several types of fractal indices will be calculated in order to address various forms of change, which are described below. More details of the sampling process and experimental setup in Appendix 1: Figures A1 and A2 are described.

### 2.2 Movement Data and Variables Acquisition

To quantify the directional structure of movement, FDI as a proxy for path complexity was used. We assessed locomotor performance in mayfly nymphs under three temperature treatments using a series of video‐based trajectory metrics. Movement trajectories were extracted from 270 videos in parallel from 90 nymphs for each family, in all thermal treatments, similar to experimental setups performed by Machuca‐Sepúlveda et al. (2025); and Shokri et al. (2021). For each individual, were quantified: Mean speed (cm/s), Maximum speed reached (MSR, cm/s), Total path length (TPL, cm), and FDI index. FDI used three independent methods: the radial method (FDIr), box‐counting (FDIb), and dilation (FDId), each capturing geometric properties of movement at different spatial resolutions. Each index captures different geometric aspects of movement: FDIr emphasizes radial dispersion, FDIb reflects area‐filling patterns, and FDId estimates dilation‐based spatial complexity (Cui and Wang 2025). In image processing, FDIr assesses the mass‐scale relation, often used for objects that are statistically radially symmetric (like diffusion clusters or branching patterns). It measures how the mass (number of pixels) enclosed in a circle (radius r) grows as the radius increases. FDIb directly measures how many n‐dimensional cubes (boxes) of a specific size are needed to completely cover the object. FDId measures the complexity based on the expansion of the object's area (or volume) as its boundary is smoothed and enlarged by a structuring element (Ai et al. 2014). For example, this last index, based on morphological dilation to measure the “thickness” of the boundary (the area surrounding the object at varying scales), have as a primary focus the surface roughness. Measuring the area V(*r*) of a dilated object using a structuring element of radius *r* (scale parameter), is suited for filled objects, surfaces, and textures. In other words, FDI are empirical techniques used to estimate the Minkowski‐Bouligand dimension, employing different strategies to measure how the object's extent changes with the scale of the measuring procedure (Radhakrishnan et al. 2004). FDIr was used as a proxy for movement complexity, with values approaching 2 indicating highly tortuous and isotropic patterns in two‐dimensional space (Benhamou 2004; Nams 2006). Missing data points were retained as NA values and excluded on a per‐variable basis during statistical analysis. The main software used in the acquisition of all these variables was ImageJ/Fiji (Schindelin et al. 2012). Through *Animal tracker* plugin (Gulyás et al. 2016), in water maze model all the body area (blob) by grayscale thresholder filter, trajectories were estimated. Videos with > 600 s, 696 × 392 pixels/8‐bit/3.8 Gb of resolution, and frames > 15,000 (segments every 1500 frames, thus forming an average of 10 segments per video) were selected. Not centroid, all the body area was used for each specimen for tracking errors, which automatically were avoided by water maze model. Software Fractalyse V3.0–0.9.1 served as a complement to calculate the fractal indices through the tracks generated by ImageJ/Fiji. Morphological data were also recorded, reduced to maximum body length (cm) and lyophilized weight (mg). Maximum body length is the longest linear proportion of each organism and lyophilized weight, provided by lyophilization process, have not volatile carbon loss by evaporation adequate for estimate energy consumption (Machuca‐Sepúlveda et al. 2022).

### 2.3 Statistical Analysis

To assess if thermal stress produces less complexity in nymphs' locomotion through FDI methods (H1), we analyzed the three types of fractal dimension extracted from the bidimensional movement paths, FDIr, FDIb, and FDId. Prior to visualization, all three FDI values were reshaped into long format or tabulation (one column indicates the measured variable, while another column records its value), with corresponding identifiers for treatment level (control, +5°C, and +10°C) and FDI type. Data were filtered to exclude missing values, and each observation retained its original pairing with thermal treatment. This graphical approach allowed direct comparison of how movement complexity (as measured by FDI) changes across increasing temperature conditions, confirmed by one‐way analysis of variances and Levene's test for unequal variances. Values were compared across three treatments between two mayfly families (Baetidae and Leptophlebiidae). The data were visualized using boxplots with overlaid jittered points to display individual variation.

To assess how microhabitat structure modulates thermal effects through FDI variation (H2), we constructed a set of candidate Generalized linear mixed‐effects models (GLMMs) for each FDI (FDIr, FDIb, and FDId). Models included combinations of fixed effects (treatment, family, and microhabitat—boulders, gravels, macrophytes, detritus, pebbles, and sand)., including “site” as a random intercept for spatial autocorrelation, included for the site of origin to account for nested spatial structure. The model with the lowest Akaike Information Criterion (AIC) was selected as the best‐fitting model for each FDI method. The relative explanatory power of treatments, taxonomy, and habitat‐related factors on movement complexity was evaluated with this approach. Furthermore, the resulting data were visualized using point and error bar plots grouped by treatment along the *x*‐axis. Lastly, the complexity of movement trajectories under thermal stress was analyzed by changes in the FDIr. Individuals were grouped by microhabitat and treatments, and distributions visualized using boxplots.

Later, Principal Component Analysis (PCA) on three fractal dimension metrics (FDIr, FDIb, FDId) to visualize clustering by family and microhabitat type—a common method in ecological studies to reduce dimensionality and detect habitat‐use patterns—was conducted (Shlens 2014). Ellipses representing 95% confidence intervals were drawn around these groups to visualize potential clustering by dominant complexity. Pillai's trace to test for overall differences in fractal dimensions across families and microhabitats was applied, supported by a Multivariate Analysis of Variance (MANOVA). Pillai's trace was chosen for its robustness to violations of multivariate normality assumptions (Rencher and Christensen 2012; Tabachnick and Fidell 2013). Univariate ANOVAs, followed by post hoc Tukey's Honest Significant Difference (HSD) test, to identify which fractal metrics and taxonomic factors were driving any significant multivariate differences, was performed. This stepwise framework aligns with best practices in studies linking habitat structure and invertebrate assemblages (Dibble and Thomaz 2009).

To examine how movement‐related and morphological traits are related to fractal habitat complexity (H3), a redundancy analysis (RDA) was executed as a central analysis. Analogous to a constrained PCA, RDA allows the joint ordination of individuals and variables in a common space. The input matrix included three fractal dimension metrics (FDIr, FDIb, and FDId), along with five morphological traits (Mean speed, MSR, TPL, Maximum body length, and Lyophilized weight). All variables were standardized (*Z*‐scores), and families were included as a grouping variable for visualization. Individual scores colored by family, along with variable vectors and 68% confidence ellipses to visually assess family‐level traits associated with habitat complexity, were plotted. This multivariate strategy is commonly used to explore functional ecological traits in heterogeneous environments (Legendre and Legendre 2012; Seuront 2011); (Legendre and Legendre 2012; Seuront 2011). To explore pairwise relationships between fractal habitat structure and functional traits, Pearson's correlation coefficients (*r*) and corresponding *p*‐values were calculated. We included three fractal dimensions (FDIr, FDIb, and FDId) along with mean speed, MSR, TPL, maximum body length, and lyophilized weight. All variables were converted to numeric, checked for completeness, and any rows containing missing values were removed prior to analysis.

All analyses and visualizations were performed using the R environment (v4.5.1). For H1, boxplots were generated using *ggplot2*, where FDI values were plotted on the *y*‐axis and treatments on the *x*‐axis, with the support of the *pivot_longer* function from the *tidyr* package. Each FDI type was shown as a distinct color within treatment groups using fill esthetics, and point‐level distributions were overlaid with the *geom_jitter* function to visualize individual data points. For H2, model selection was performed using AIC, and models were ranked using the *MuMIn* package. Predictions from the models were obtained with the *ggeffects* package and visualized with *ggplot2*, faceting by family and dimension type, and coloring by microhabitat. Other packages used were *tidyverse* and *ggplot2*. The PCA was performed using the *prcomp* function, and the first two components were retained for visualization. All statistical analyses were performed with the *emmeans* package, and predicted group means with 95% confidence intervals were visualized using *ggplot2*. Predicted marginal means and 95% confidence intervals were extracted using the *ggpredict* function from the *ggeffects* package. In the case of H3, the packages of the R environment were chiefly *vegan*. Also, the *rcorr* function from the Hmisc package was used. Correlation results were visualized using a *ggplot2* heatmap with *geom_tile* and *geom_text* functions, where each cell displayed both the correlation coefficient and the exact *p*‐value in the format “*r* = 0.45, *p* = 0.012,” for example.

## 3 Results

### 3.1 Warming Decreases Movement Complexity

**FIGURE 1:**
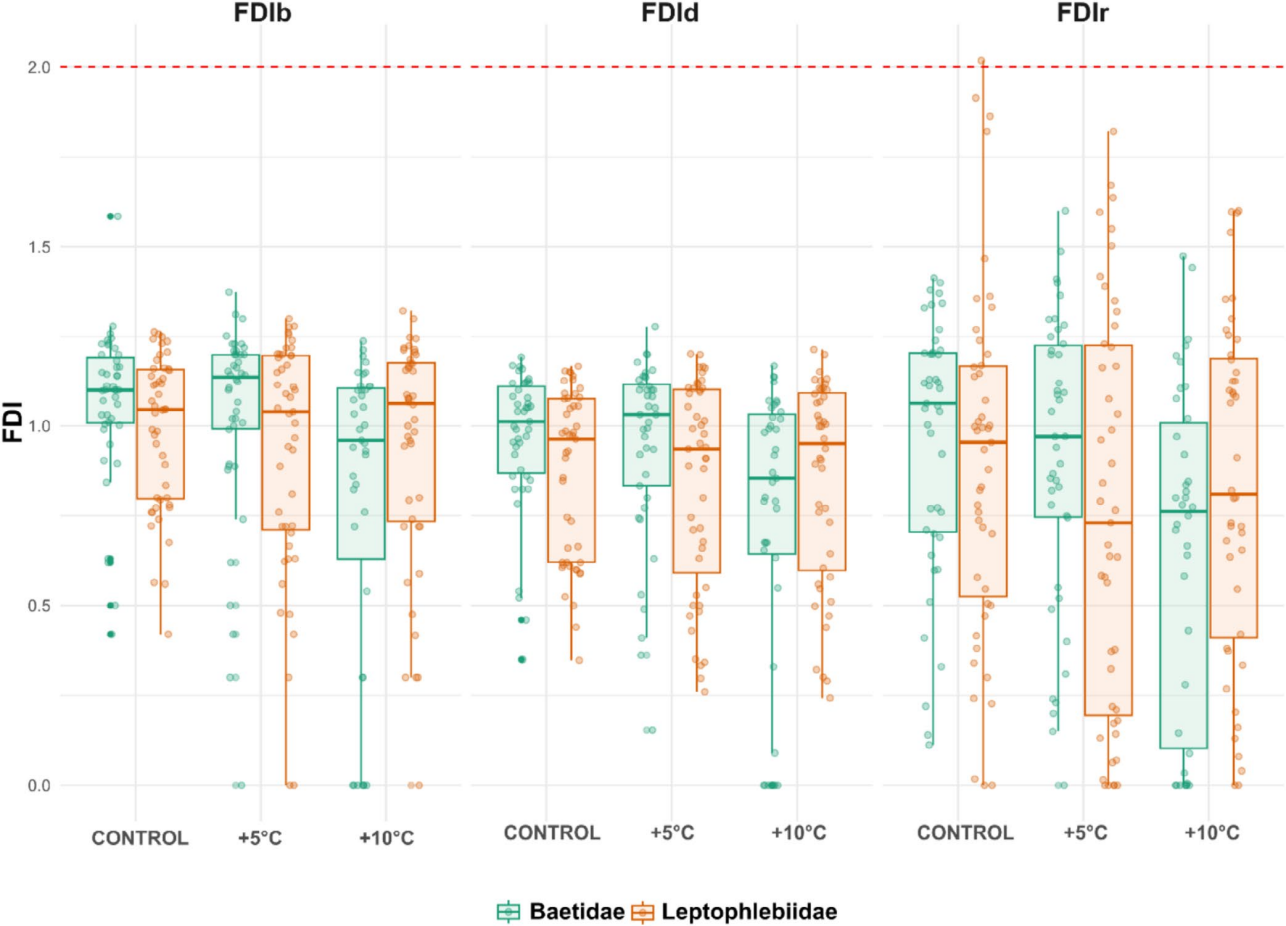
FDI of trajectories estimated using three geometric methods—box‐counting (FDIb), dilation (FDId), and radial (DFIr)—for mayfly nymphs from the families Baetidae and Leptophlebiidae under three water temperature treatments: Control, +5°C, and +10°C. The dashed red line indicates *D* = 2, corresponding to perfectly isotropic two‐dimensional movement.

FDI exhibited differential responses across thermal treatments (Figure 1). While median values remained relatively stable, increased temperature treatments (+5°C and +10°C) resulted in a noticed variability in FDI values, particularly in the radial method (FDIr). Nonetheless, FDIr (*p* < 0.05) variances can be considered homogeneous according to Levene's test (*p* > 0.05), in distinction to both FDIb (*p* < 0.01) and FDId (*p* < 0.05), with significative differences in Levene's test results (*p* < 0.001). This pattern suggests that thermal stress shapes the structure of movement trajectories, likely reducing their spatial complexity.

FDI of paths varied with the estimation method, thermal treatment, and taxonomic identity. In general, values of FDI derived from the FDIr method were lower than those estimated via FDIb or FDId, particularly in Baetidae. For this family, FDI values increased moderately with rising temperature, approaching the theoretical isotropic threshold (*D* = 2) under the highest thermal stress condition. In contrast, Leptophlebiidae exhibited consistently broad FDI values across treatments, showing higher variation and decreases as thermal stress increases. Although this ambivalence produces a contradiction with respect to H1, there is partial evidence of a decrease in FDI as heat stress increases in terms of one of the families.

### 3.2 Microhabitat Structure Modulates Thermal Effects

Model selection results revealed that the most parsimonious models differed across FDI according to the lowest AIC values. For FDIb, FDIr, and FDId, the best model included mostly treatment as a predictor (+10°C), suggesting a strong and independent effect of thermal stress on complexity movement. Nonetheless, some models with the lowest AIC included treatment and microhabitat, indicating that physical configuration could modulate the effect of temperature, highlighting divergent responses depending on the habitat in which nymphs displace (Table 1).

**TABLE 1:** Model selection results based on Akaike Information Criterion (AIC) for mixed‐effects models assessing the effects of family identity, microhabitat (sand and detritus [detr]), and thermal treatment (+5°C and 10°C), and their fixed effects on different fractal dimensions of mayfly movement behavior.

Model	Fixed effects (best model fitted)	Estimate ± SE	*p*	AIC	ΔAIC	Weight
FDIb1	Treatment: +5°C, micro_habitat:detr	−0.593 ± 0.281	0.035	350.004	0	0.980
FDIb2	Treatment: +10°C	−0.171 ± 0.038	1.27E‐05	357.820	7.815	0.019
FDIb3	Treatment: +10°C	−0.278 ± 0.054	7.73E‐07	368.170	18.165	0.0001
FDIb4	Treatment: +10°C	−0.172 ± 0.038	1.51E‐05	404.393	54.389	1.52E‐12
FDIr1	Micro_habitat:sand	−0.497 ± 0.270	0.073	79.351	0	0.995
FDIr2	Treatment: +10°C	−0.188 ± 0.068	0.006	90.256	10.904	0.004
FDIr3	Treatment: +10°C	−0.300 ± 0.098	0.002	96.709	17.358	0.0001
FDIr4	Treatment: +10°C	−0.187 ± 0.068	0.006	145.088	65.737	5.29E‐15
FDId1	Treatment: +5°C, micro_habitat:detr	−0.428 ± 0.249	0.086	9.945		0.993
FDId2	Treatment: +10°C	−0.133 ± 0.033	9.02E‐05	19.999	10.053	0.006
FDId3	Treatment: +10°C	−0.234 ± 0.047	1.43E‐06	28.081	18.136	0.0001
FDId4	Treatment: +10°C	−0.133 ± 0.033	0.0001	97.102	87.156	1.18E‐19

*Note:* Models are ranked by increasing AIC values, with the lowest AIC indicating the best‐supported model. ΔAIC (delta) values represent the difference from the best model, show estimate ± SE, *p*‐values, and weights that indicate the relative likelihood of each model given the data.

**FIGURE 2:**
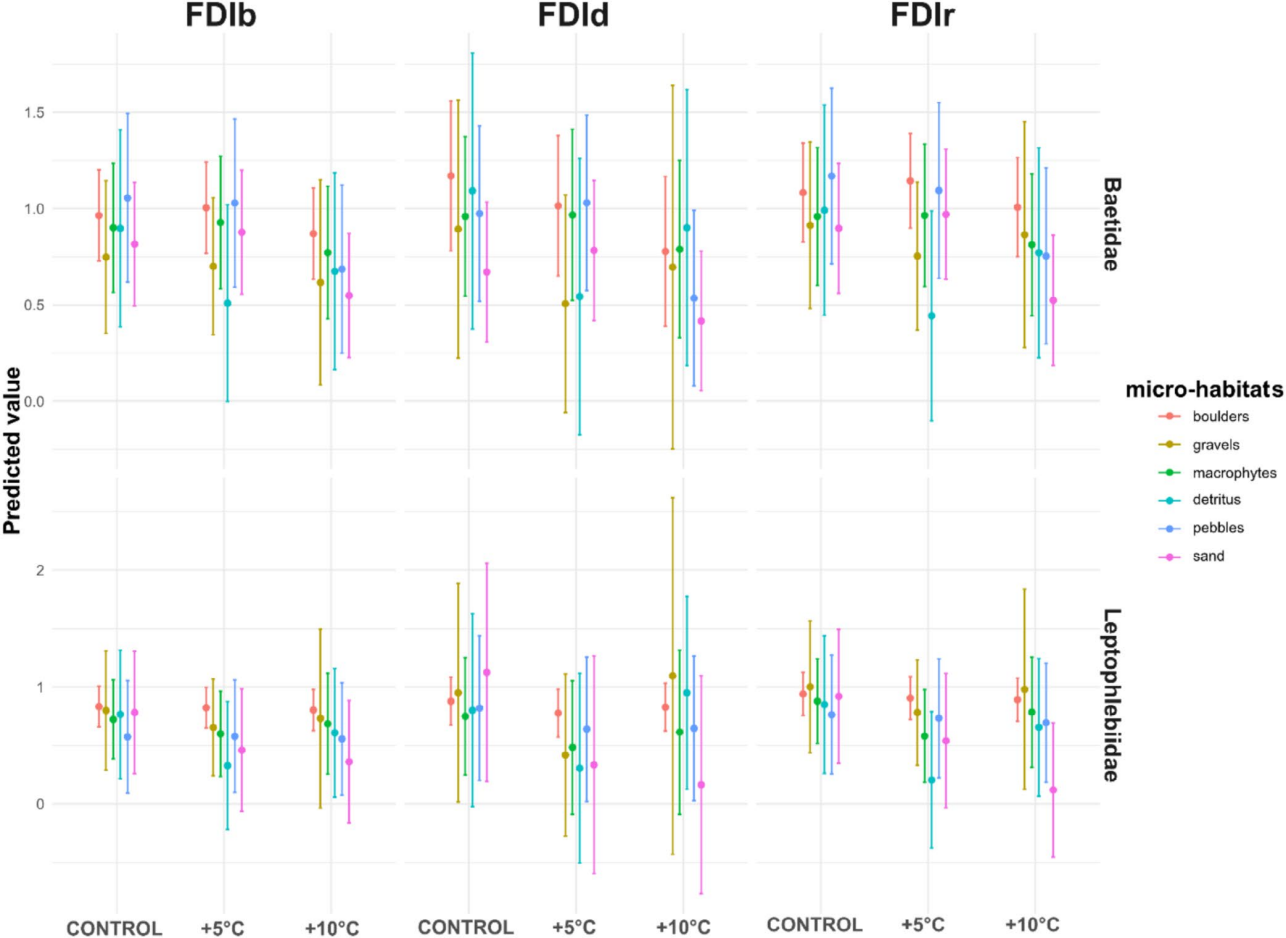
Predicted effects of treatments on three fractal dimension indices (FDIb, FDId, and FDIr) in nymphs from the families Baetidae and Leptophlebiidae, across different microhabitats. Lines represent model‐based estimates from generalized linear mixed models (GLMM), and shaded areas indicate 95% confidence intervals.

GLMMs fitted for each FDI revealed distinct responses to thermal stress throughout their predicted values (Figure 2). The FDId showed divergent trends between families, with Baetidae being more susceptible to temperature increases, particularly in open microhabitats. For the FDIb, treatment‐related changes were subtler, although a slight decline in fractal complexity under elevated temperatures is evident. The FDIr displayed relatively stable values across treatments, especially in Leptophlebiidae. Overall, these findings suggest that the spatial complexity of movement is affected by temperature increase. However, the magnitude and direction of such effects depend on the estimation method, family, and microhabitat. Indeed, across all three estimation methods, FDI showed a consistent decrease with increasing thermal stress (from control to +10°C treatment). This pattern was consistent across Ephemeroptera families and microhabitats, although the magnitude of reduction varied.

**FIGURE 3:**
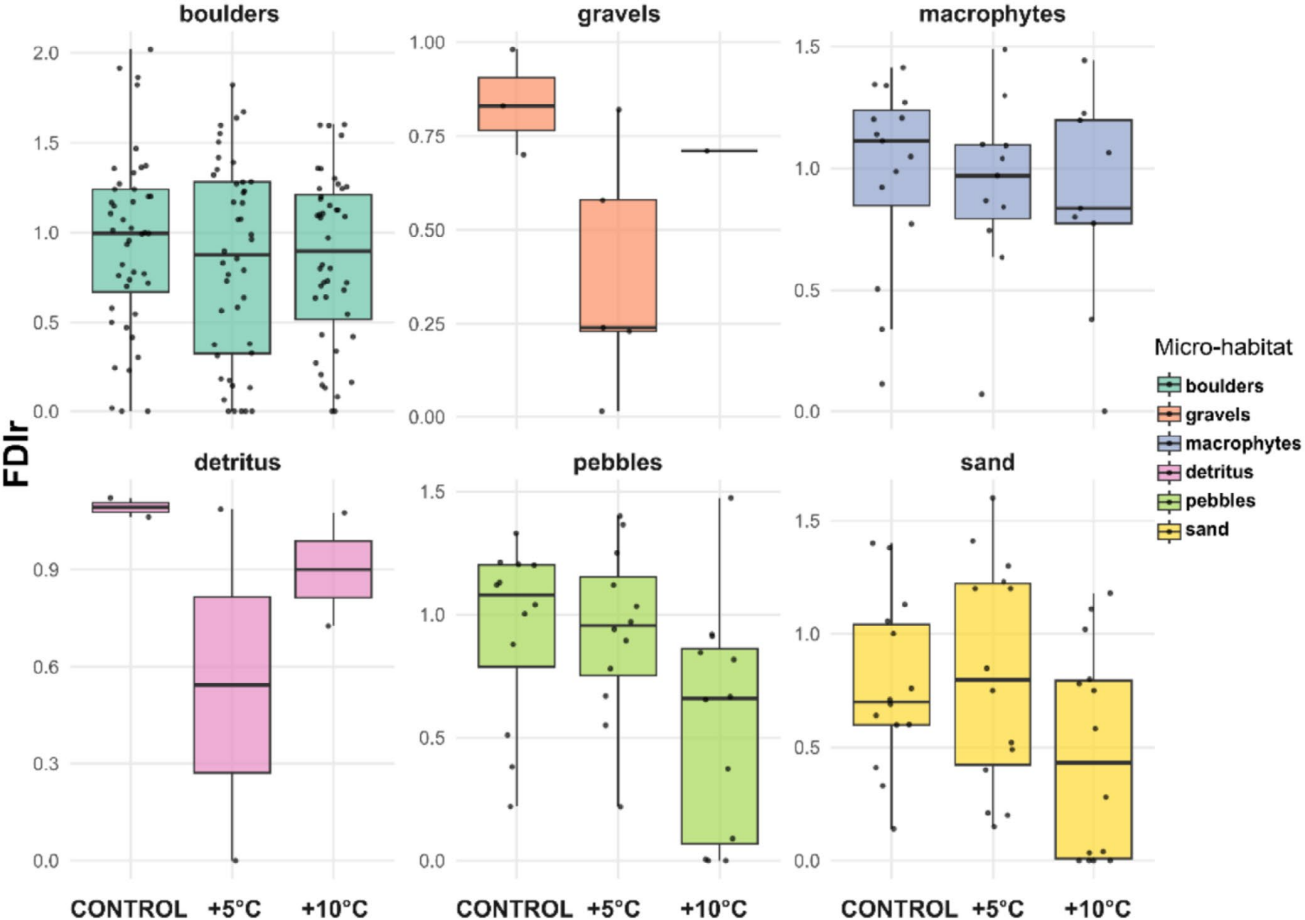
Boxplots of FDIr across thermal treatments (control, +5°C, and +10°C) and grouped by microhabitat types (boulders, gravels, macrophytes, detritus, pebbles, and sand). Higher FDIr values indicate more complex locomotor patterns.

Due to logistics, accessibility, and availability of microhabitats, inequal number of specimens in each microhabitat were acquired. Therefore, descriptive results are presented here with respective percentages of specimens used in treatments: 50.76% (boulders), 3.44% (gravels), 13.74% (macrophytes), 2.29% (detritus), 13.74% (pebbles), and 16.03% (sand). The response of FDIr to thermal stress varied significantly across microhabitats (Figure 3). A modulation effect is evident as an informative example, with microhabitat as the origin of nymphs influencing the degree of complexity retained under increasing thermal stress. The boxplots revealed a potential modulation pattern, where insects from structurally complex habitats, for example, macrophytes or boulders, tended to maintain higher FDI values across treatments showing no significant differences (one‐way ANOVA: *F* = 0.14; *p* = 0.87 and *F* = 0.3; *p* = 0.7382, respectively). Compared to those from bidimensional microhabitats, for example, pebbles or sand, whose FDI values declined more steeply under warming with more significance (*F* = 3.39; *p* < 0.01 and *F* = 3.36; *p* ≤ 0.01, respectively). Hence, under +10°C treatment, the reduction in FDI was more pronounced in organisms from microhabitats such as pebbles and sand, denoting a higher trend to less complex behavior.

**FIGURE 4:**
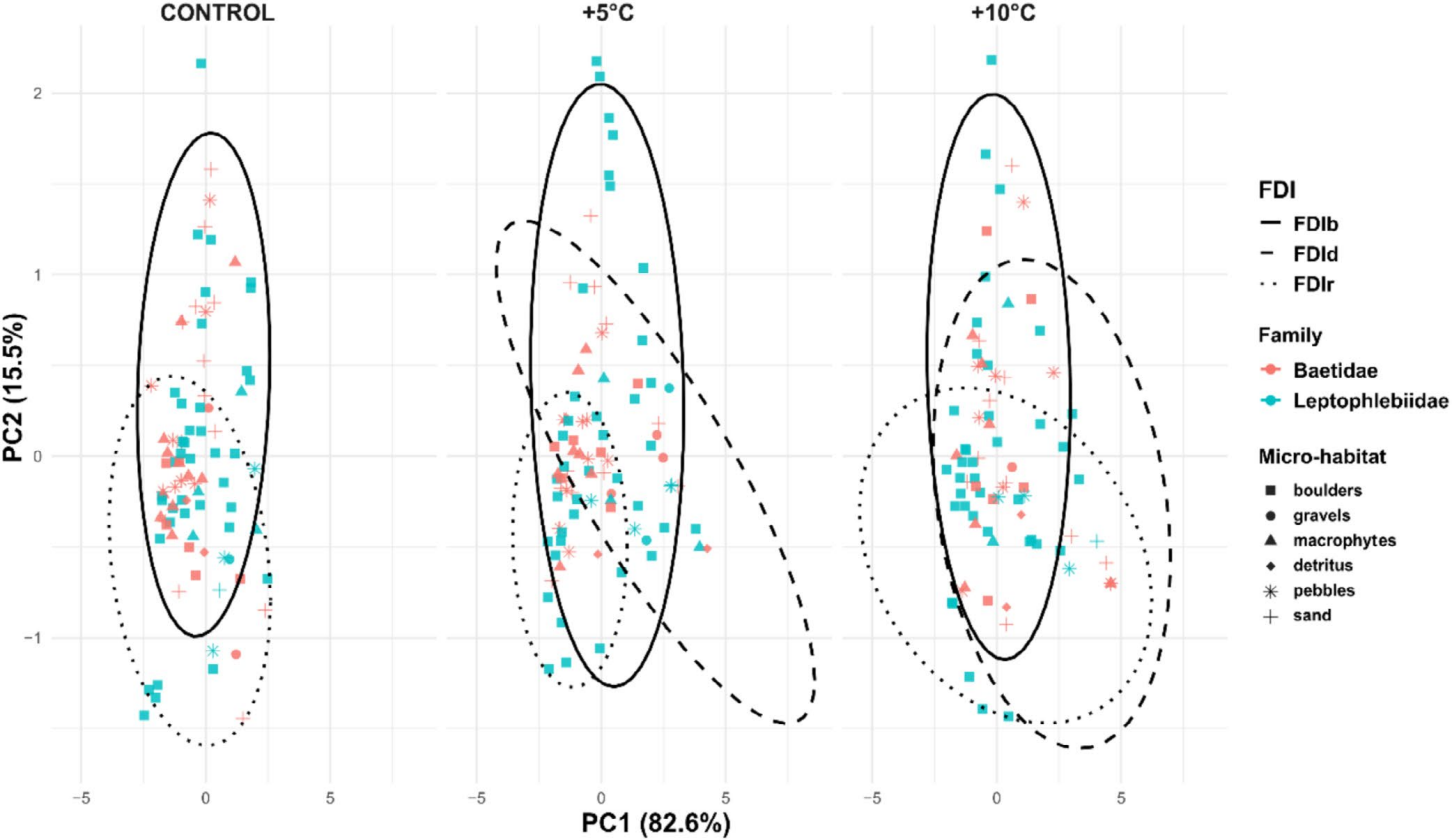
Principal Component Analysis (PCA) of fractal dimension metrics (FDIr, FDIb, and FDId) in mayfly nymphs subjected to thermal stress. Each point represents an individual, colored by family (Baetidae in pink, Leptophlebiidae in blue) and shaped by microhabitat where was captured. Ellipses represent 95% confidence intervals around groups sharing the same dominant fractal dimension (i.e., the highest FDI value among the three metrics). The first two principal components explained 82.6% and 15.5% of the total variance, respectively.

The PCA revealed clear structuring of individuals in relation to their dominant fractal dimensions and treatment (Figure 4). Across treatments, individuals with similar dominant dimensions tended to cluster together in PCA space, particularly those with high FDIr or FDIb. In the control treatment, only FDIr and FDIb emerged as dominant dimensions, while FDId was absent, suggesting a lower habitat dilation complexity in that condition. In contrast, both +5°C and + 10°C displayed individuals distributed across all fractal dimension groups, indicating more heterogeneous microhabitat structure under thermal stress. PC1 and PC2 accounted for a combined 98.1% of total variance, with PC1 primarily differentiating individuals by their FDI level. While Leptophlebiidae individuals were distributed more broadly along PC1 and PC2, Baetidae tended to cluster in specific regions of the plot (excluding +10°C treatment).

Complementary with PCA, in order to test the effect of family and microhabitat on the three fractal dimension metrics, MANOVA was performed. The Pillai's trace statistic revealed a multivariate effect of microhabitats (Pillai = 0.150, *F* = 2.675, *p* < 0.001) more than family (Pillai = 0.012, *F* = 1.031, *p* < 0.377). Pillai's trace of family is lower than microhabitats, indicating that the combination of FDIr, FDIb, and FDId has stronger evidence in the microhabitat examined. Thermal treatment also modulated FDI: while control and +5°C treatments did not differ significantly in most pairwise comparisons, a general trend toward lower FDI under extreme warming (+10°C) was observed, especially in microhabitats like pebbles and sandThe post hoc Tukey HSD analysis revealed significant differences in FDI values across several factor combinations. Among microhabitats, detritus and boulders, supported consistently higher FDI values compared to sand and gravel microhabitats, with multiple pairwise comparisons, reaching statistical significance (e.g., FDId: macrophytes—gravels, *p* < 0.01) (Table 2).

**TABLE 2:** Post hoc Tukey HSD pairwise comparisons for fractal dimension methods (FDIr, FDIb, and FDId) across different families and microhabitats.

Comparison	diff	lwr	upr	*p* adj	FDI
Leptophlebiidae‐Baetidae	−0.017	−0.132	0.096	0.756	FDIr
Gravels‐boulders	−0.321	−0.782	0.140	0.344	
Macrophytes‐boulders	0.040	−0.214	0.295	0.997	
Detritus‐boulders	−0.048	−0.608	0.510	0.999	
Pebbles‐boulders	−0.079	−0.331	0.172	0.944	
Sand‐boulders	−0.204	−0.441	0.032	0.135	
Macrophytes‐gravels	0.362	−0.138	0.862	0.302	
Detritus‐gravels	0.272	−0.433	0.978	0.877	
Pebbles‐gravels	0.241	−0.257	0.741	0.732	
Sand‐gravels	0.116	−0.375	0.609	0.983	
Detritus‐macrophytes	−0.089	−0.681	0.502	0.998	
Pebbles‐macrophytes	−0.120	−0.438	0.197	0.886	
Sand‐macrophytes	−0.245	−0.551	0.061	0.199	
Pebbles‐detritus	−0.030	−0.621	0.559	0.999	
Sand‐detritus	−0.155	−0.740	0.429	0.973	
Sand‐pebbles	−0.124	−0.429	0.179	0.846	
Leptophlebiidae‐Baetidae	−0.013	−0.087	0.059	0.7139	FDIb
Gravels‐boulders	−0.325	−0.622	−0.029	**0.022**	
Macrophytes‐boulders	0.008	−0.155	0.172	0.999	
Detritus‐boulders	−0.280	−0.640	0.078	0.222	
Pebbles‐boulders	−0.076	−0.238	0.085	0.758	
Sand‐boulders	−0.168	−0.321	−0.015	**0.020**	
Macrophytes‐gravels	0.334	0.012	0.656	**0.036**	
Detritus‐gravels	0.045	−0.409	0.499	0.999	
Pebbles‐gravels	0.249	−0.071	0.571	0.225	
Sand‐gravels	0.157	−0.159	0.473	0.709	
Detritus‐macrophytes	−0.289	−0.670	0.091	0.249	
Pebbles‐macrophytes	−0.084	−0.289	0.119	0.842	
Sand‐macrophytes	−0.177	−0.374	0.020	0.106	
Pebbles‐detritus	0.204	−0.175	0.584	0.633	
Sand‐detritus	0.112	−0.263	0.488	0.955	
Sand‐pebbles	−0.092	−0.288	0.103	0.752	
Leptophlebiidae‐Baetidae	−0.031	−0.098	0.034	0.346	FDId
Gravels‐boulders	−0.333	−0.603	−0.063	**0.006**	
Macrophytes‐boulders	0.014	−0.134	0.163	0.999	
Detritus‐boulders	−0.239	−0.565	0.087	0.289	
Pebbles‐boulders	−0.089	−0.236	0.057	0.498	
Sand‐boulders	−0.131	−0.269	0.007	0.075	
Macrophytes‐gravels	0.347	0.055	0.640	**0.009**	
Detritus‐gravels	0.094	−0.318	0.506	0.986	
Pebbles‐gravels	0.243	−0.047	0.535	0.160	
Sand‐gravels	0.202	−0.085	0.489	0.333	
Detritus‐macrophytes	−0.253	−0.599	0.092	0.287	
Pebbles‐macrophytes	−0.104	−0.289	0.081	0.592	
Sand‐macrophytes	−0.145	−0.324	0.033	0.184	
Pebbles‐detritus	0.149	−0.195	0.494	0.815	
Sand‐detritus	0.1079	−0.233	0.449	0.944	
Sand‐pebbles	−0.041	−0.219	0.136	0.985	

*Note:* Each row shows the mean difference (diff), the 95% confidence interval (lwr, upr), and the adjusted *p*‐value (*p* adj). Significant comparisons (*p* adj < 0.05) are highlighted in bold. Positive values of difference indicate higher FDI in the first group listed in the comparison column.

### 3.3 Families Vary by Locomotor and Biological Traits

**FIGURE 5:**
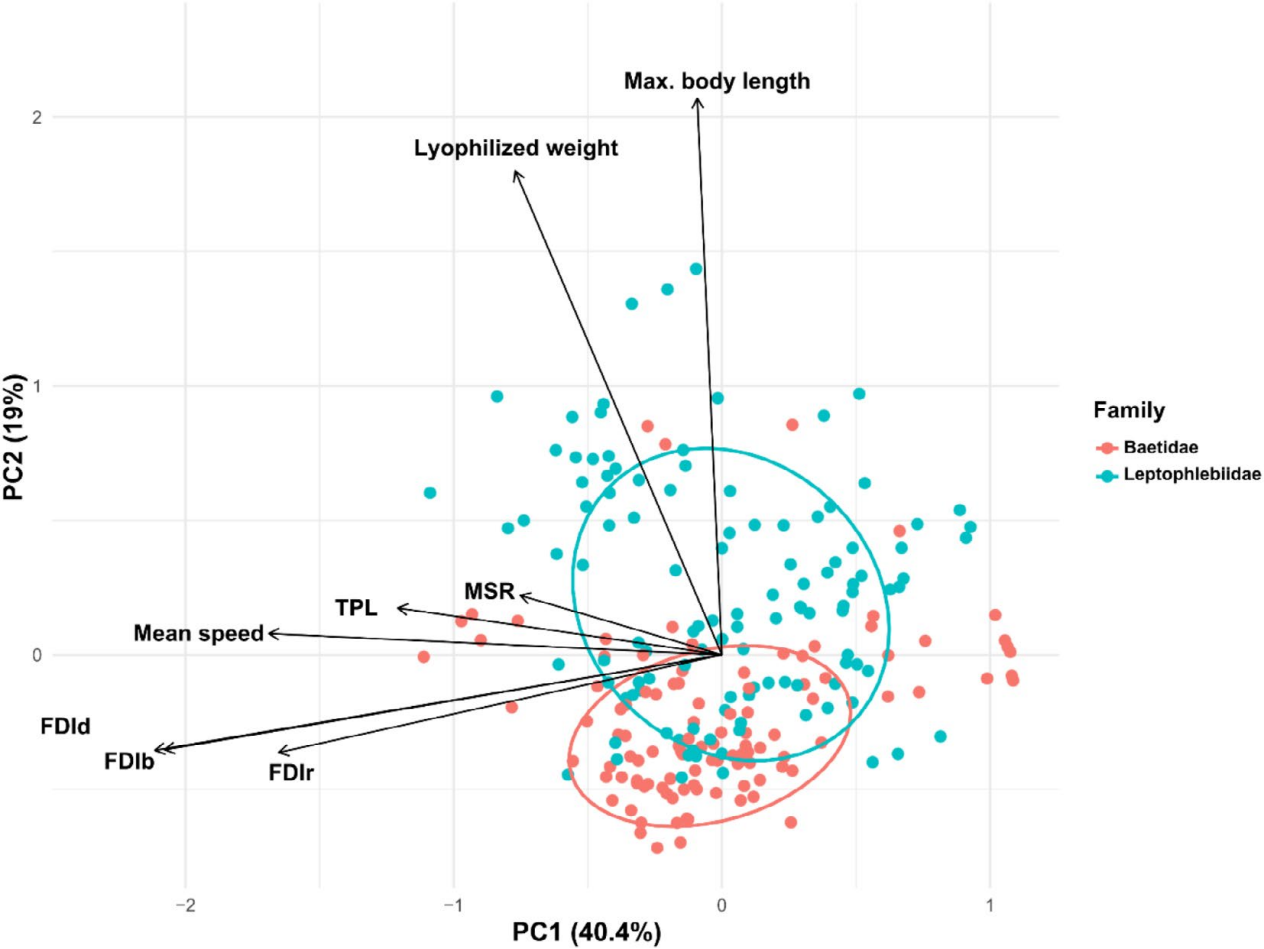
Redundancy analysis (RDA) shows the relationship between movement complexity, locomotor performance, and morphological traits. Each point represents specimens, colored by family. Arrows indicate the direction and magnitude of association for each explanatory variable: FDI (radial, box‐counting, and dilation), Mean speed, MSR, TPL, Maximum body length, and Lyophilized weight.

Even though previous analyses did not reveal clear differences between the two families, RDA revealed clear multivariate structuring in relation to both FDI metrics and biological traits. The first two axes explained a combined 59.4% of the total variance, with PC1 accounting for 40.4%. Families exhibited distinct groupings, with Baetidae associated with higher fractal complexity and locomotor performance (e.g., higher Mean speed, TPL). While Leptophlebiidae clustered closer to higher body size (Max. body length) or metabolic functioning (Lyophilized weight) (Figure 5). Vector directions showed that fractal dimensions aligned positively with mobility and size traits. Suggesting that movement complexity covaries with developing strategies that could be high displacement or speed, corresponding to Baetidae. In contrast, Leptophlebiidae have higher morphological characteristics, such as size and weight, to the detriment of the movement's performance and complexity.

**FIGURE 6:**
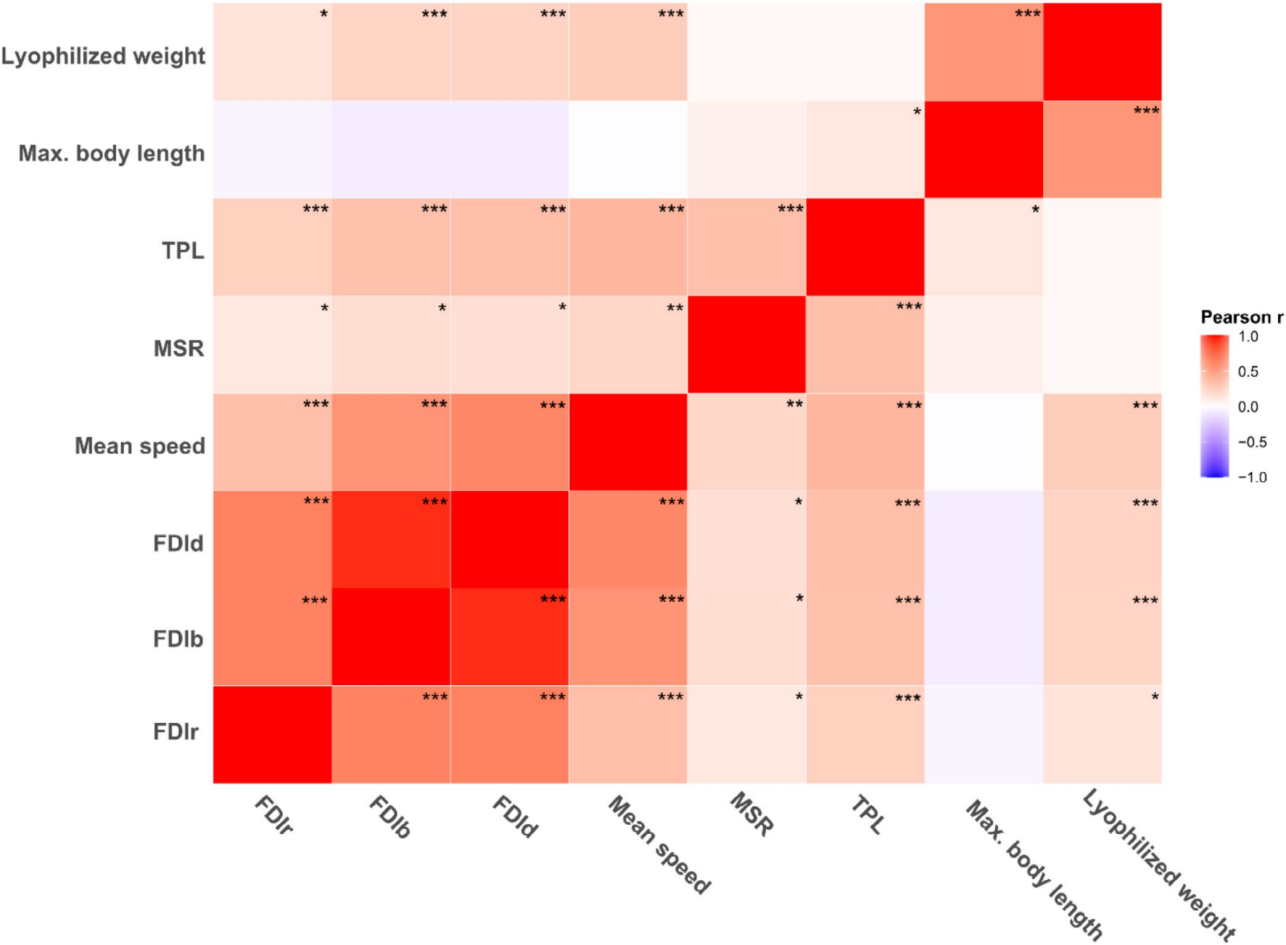
Heatmap of Pearson correlation coefficients (r) between FDI, locomotor, and morphological traits in mayfly nymphs. Each cell displays the correlation coefficient (r) and associated *p*‐value, (***p < 0.001; **p < 0.01; *p < 0.1). Warmer colors indicate positive correlations, and cooler indicate negative correlations, with intensity proportional to correlation strength. Variables were standardized and correlation analyses were based on pairwise complete observations.

Pearson correlation analyses revealed consistent and significant associations between FDI metrics and both locomotor and morphological traits. All three fractal dimensions—FDIr, FDIb, and FDId—were positively correlated with Mean speed (*r* = 0.33–0.60, *p* < 0.001), TPL, and MSR, indicating that individuals in more complex habitats tended to move faster, farther, and with less frequent pauses. Additionally, moderate correlations with Lyophilized weight (*r* ≈0.14–0.23) suggest a potential link between fractal structure and energetic or physiological investment (Figure 6). These results further support our hypothesis that movement complexity is a driver of functional trait expression in mayfly nymphs, associated with locomotor behavior.

## 4 Discussion

Our findings support the use of fractal metrics as sensitive indicators of behavioral plasticity and complexity of animal motility, under environmental stress. The observed reduction in FDI under thermal stress in most cases aligns with the hypothesis that elevated temperatures lead to less complex movement patterns, but only at the scale used in this experiment. The study reveals that reduced FDI values under warmer conditions, indicating insect trajectories became more directional and nonuniform. In 2D pathways, fractal scaling depends on orientation and direction as well, which does not support anisotropy it all (Seleznov et al. 2020). Because anisotropy, as conceived in the literature, depends on a multi‐scale evaluation with directional/angular analysis (Trotter et al. 2019), which is not the specific focus of this work. The expansion of FDI spread under the highest temperature treatment may indicate divergent behavioral strategies or physiological limits (Bodlah et al. 2023). It is important to clarify that water temperature augmentation in aquatic organisms can produce different stress responses compared to terrestrial or aerial insects, which are described below. Several studies suggest that movement dimensionality is shaped by both internal physiological traits and external environmental constraints (Getz and Saltz 2008; Nathan et al. 2008). From our results, we can only associate kinetic effects, that is, there is an upward restrictive influence of temperature on metabolism and neurophysiology. Other types of effects have been recognized in which the bottom‐up integration of thermal information intentionally initiates or modifies behavior, such as behavioral thermoregulation or thermal orientation (Abram et al. 2017). However, a greater number of response variables are needed to deduce some degree of behavioral thermoregulation, for example. Such interpretation would align with previous work indicating that elevated temperatures can restrict exploratory behavior and path complexity in this kind of aquatic invertebrates (Cui and Wang 2025; Gibert et al. 2016). The decline in fractal dimension values under +5°C and +10°C treatments, especially for FDIr and FDId, suggests that higher temperatures may constrain complex movement patterns, potentially as an energetic or behavioral adaptation. Moreover, insects from more heterogeneous or thermally buffered environments maintained higher FDI values under stress, while those from more exposed habitats showed reduced complexity (Terlau et al. 2023).

Regarding families, directional pattern was particularly pronounced in Baetidae, whose FDI values dropped more steeply compared to Leptophlebiidae. More experimental parameters could reveal taxon‐specific sensitivity to temperature. It remains for the future to study the physiological and morphological differences between Baetidae and Leptophlebiidae, which allow their inherent strategies of resistance to thermal stress. Leptophlebiidae and Baetidae seem to segregate into distinct structural niches defined by fractal habitat complexity. Nevertheless, our data also depends on the genus that compose each family, therefore, we consider that there is greater diversity if the taxonomic resolution is more refined. This shortcoming arises from the lack of more complete taxonomic records in Latin America (Cortelezzi and Paz 2023). In this case, the plan would be to develop biological trait indices together with models that relate taxa at the genus or species level to stress factors. With regard to what we collect, PCA‐based clustering agrees with findings from MANOVA and post hoc Tukey HSD analyses, which indicated significant habitat differences more than interfamily in FDI values. This supports prior work demonstrating that structural attributes of habitat strongly influence nymphs' assemblages. For instance, (Dibble and Thomaz 2009) found positive relationships between fractal complexity and invertebrate abundance and richness in macrophyte habitats. Similarly, (Williamson and Lawton 1991) showed that fractal vegetation architecture predicts arthropod body size distributions. Nonetheless, PCA results indicate that the dominant type of fractal complexity (FDIr, FDIb, or FDId) is not randomly distributed among individuals but rather reflects underlying ecological and taxonomic structure. The separation of individuals into distinct ellipsoidal clusters suggests that certain families (e.g., Baetidae) preferentially exhibit specific movement complexity patterns, possibly reflecting adaptations to habitat flow regimes or microhabitat configuration.

The variation in GLMM across FDI metrics underscores the multidimensional pattern of behavioral responses to thermal stress. The fact that treatment alone best explained radial complexity (FDIr) suggests that thermal stress imposes a broad, directional effect on movement complexity. Meanwhile, the inclusion of family and interaction terms in the significative models for FDIb and FDId indicates that taxonomic traits modulate how organisms navigate structurally intricate environments. The observed pattern—where individuals from complex habitats sustained higher FDI values across increasing temperatures—suggests that structural heterogeneity may buffer the impacts of thermal stress on behavioral traits. Similar patterns have been reported in studies where environmental complexity contributes to functional resilience under abiotic stressors (Terlau et al. 2023; With 1994). In contrast, organisms from simpler habitats displayed reduced complexity under warming, possibly reflecting limitations in navigational or exploratory behavior in thermally challenging environments. Habitats such as gravel and detritus, typically characterized by finer and more fragmented, showed strong reductions in movement complexity, likely reflecting thermal impairment of fine‐scale navigation or changes in local flow dynamics (Loke and Chisholm 2022). That modulation pattern reinforces the significance of microhabitats in spatial ecology for relocation or dispersion contexts, taking into account a dynamic related to interconnected patching (Banet et al. 2022).

In the overhead view, organisms change their body posture during the videos (taking frontal positions in 1D or longitudinal in 2D with respect to the camera), which is estimated by FDId. FDId yields lower values in Baetidae in the higher temperature treatment. This suggests several characteristics, such as a higher locomotor activity at a point rather than following a route. It would be necessary to analyze in more detail the movement behaviors of both families in a 2D plane, considering the vertical movement tracks in the water column. Now, if we take this feature to the physical structure of microhabitats offer structural complexity to refuge dispersion due hydric connectivity and flow, likely appeared to promote movement behaviors in one point or in a trajectory (Frakes et al. 2021). Conversely, lower FDI in open and unstable microhabitats such as sand and gravel, may reflect more linear or escape‐type movements, especially under elevated temperatures. These findings align with prior work highlighting the interaction between microhabitat complexity, and thermal stress in shaping invertebrate behavioral plasticity (Seebacher et al. 2015). Importantly, the shift in FDI patterns under +10°C treatment hints a threshold‐like thermal response. Where excessive warming disrupts fine‐scale spatial exploration, potentially impacting ecological roles such as resource acquisition or predator avoidance (Vucic‐Pestic et al. 2011). The lack of consistent patterns in macrophytes and boulders may stem from structural heterogeneity buffering thermal impacts. These microhabitat‐specific trajectories suggest that future climate scenarios may selectively alter the functional role of species through their spatial behavior, emphasizing the need to integrate behavioral metrics in conservation‐oriented managements (Youngsteadt et al. 2023).

The PCA results support the fact that thermal regimes influence the spatial organization of mayfly nymphs through shifts in habitat structural complexity. The absence of FDId‐dominated individuals in control treatment may reflect a more homogeneous environment with fewer transitional or irregular habitat elements typical of undisturbed, cooler streams. In contrast, +5°C and + 10°C treatments, included individuals across all three dominant fractal dimensions, suggesting that thermal stress can promote microhabitat heterogeneity. These results are associated with the broader ecological principle that increased habitat complexity allows for greater trait‐based partitioning (Srednick et al. 2023; Ward et al. 2023). In this case, FDI serve not only as quantifiable descriptors of microhabitat structure, but also as predictive variables for understanding species–habitat interactions across environmental gradients. Both intrinsic (taxonomic) and extrinsic (habitat) factors are definitely the main drivers to thermal modulation of movement complexity, in agreement with findings from stress ecology and behavioral plasticity studies (e.g., Dell et al. 2011; Seebacher and Franklin 2012).

The outcomes support that structurally complex habitats shapes locomotor and morphological traits, which can be extracted visually from RDA analysis and heatmap correlation. In RDA, observed multivariate patterns suggest that families segregate along structural and functional gradients, consistent with ecological filtering and niche differentiation. High fractal habitats may select taxa with enhanced movement efficiency and greater weight, possibly due to increased surface area, flow turbulence, or shelter opportunities. These results are side with previous research linking physical habitat heterogeneity to functional diversity and invertebrate performance (Dibble and Thomaz 2009; Morse et al. 1985; Seuront 2011). Additionally, habitat complexity is known to influence community composition and functional diversity in aquatic taxa (Ward et al. 2023). Moreover, the use of RDA revealing coordinated trait responses to environmental structure, which is increasingly recognized in movement ecology (Nathan et al. 2008). The correlation matrix reveals robust associations between habitat structure and functional traits. Notably, FDId and FDIb exhibit strong positive correlations with locomotion metrics such as Mean speed, MSR, and TPL (r up to 0.60, *p* < 0.001). Likely due to comfortable refugia and navigation cues—a pattern consistent with ecological theory on structural habitat effects, supports the concept that increased microhabitat complexity enhances nymphs' mobility and exploration capacity (Mocq et al. 2021). Our findings lend further mechanistic insight into the multivariate patterns observed in the PCA and RDA analyses, reinforcing the role of fractal habitat structure as a driver not only of trait variation, but also possibly for ecological niche differentiation.

## 5 Conclusions

In summary, our research provides consistent evidence that the movement complexity of mayfly nymphs is determined by a combination of thermal stress, habitat structure, and taxonomy. With integrated univariate, multivariate, and mixed‐effects statistical analysis, fractal locomotion metrics help measure this. The initial hypothesis (H1) received partial support. Elevated temperatures were associated with a reduction in FDIr and FDId in all treatments, especially in the most stressful (+10°C). These patterns imply a consistent directional bias in movement trajectories, consistent with reduced behavior under physiological stress and progressive simplification. The importance of this reaction varied among fractal indices, underscoring that different dimensions capture complementary facets of locomotor organization rather than redundant data. The analyses provided strong support for the second hypothesis (H2). Post hoc comparisons and mixed‐effects models showed strong interactions between treatments and microhabitat, indicating that habitat context modifies how thermal stress reduces movement complexity. More two‐dimensional microhabitats (e.g., sand and pebbles) showed strong decreases in FDI. These results emphasize the importance of physical structure in preserving functional movement patterns and the response of taxa living in such microhabitats. It is suggested that habitat heterogeneity may reduce behavioral degradation in thermal stress scenarios. Our third hypothesis (H3) was also confirmed through multivariate analyses (MANOVA and PCA), which showed a marked response at the family level in fractal indices, given that Baetidae and Leptophlebiidae are found in different areas of the multivariate space. Consistent with different ecological strategies, these variations imply that taxonomic identity constrains how movement behavior is organized in various habitat types and thermal circumstances. Depending on their locomotor flexibility and structural niche, the responses recorded suggest that taxa may suffer disproportionately from warming and habitat loss. Taking together, our results show that thermal stress affects not only the intensity but also the structure of aquatic insect movement. Ultimately, this study contributes to the mechanistic understanding of how habitat degradation and global warming can interact to reduce behavioral complexity in freshwater habitats.

## Author Contributions

**Jorge Machuca‐Sepúlveda:** conceptualization (equal), data curation (equal), formal analysis (equal), methodology (equal), project administration (equal), resources (equal), software (equal), visualization (equal), writing – original draft (equal), writing – review and editing (equal). **Mauricio Zamorano:** supervision (equal), validation (equal), writing – review and editing (equal). **Jorge G. Farías:** funding acquisition (equal), investigation (equal), project administration (equal).

## Funding

This research was funded by ANID (Agencia Nacional de Investigación y Desarrollo) scholarship, grant number 21210601.

## Ethics Statement

The authors have nothing to report.

## Consent

The authors have nothing to report.

## Conflicts of Interest

The authors declare no conflicts of interest.

## Data Availability

Dataset and R code are in the repository: https://github.com/cerebrodoce/Fractal‐indices‐as‐estimators‐of‐the‐complexity‐of‐Ephemeroptera‐nymph‐movement.git.
